# Muscle Physiology Changes Induced by Every Other Day Feeding and Endurance Exercise in Mice: Effects on Physical Performance

**DOI:** 10.1371/journal.pone.0013900

**Published:** 2010-11-09

**Authors:** Elizabeth Rodríguez-Bies, Sara Santa-Cruz Calvo, Ángela Fontán-Lozano, José Peña Amaro, Francisco J. Berral de la Rosa, Ángel M. Carrión, Plácido Navas, Guillermo López-Lluch

**Affiliations:** 1 Centro Andaluz de Biología del Desarrollo (CABD), Universidad Pablo de Olavide-CSIC, CIBERER-Instituto de Salud Carlos III, Sevilla, Spain; 2 División de Neurociencia, Departamento de Fisiología, Anatomía y Biología Celular, Universidad Pablo de Olavide, Sevilla, Spain; 3 Departamento de Ciencias Morfológicas, Facultad de Medicina, Universidad de Córdoba, Córdoba, Spain; 4 Departamento de Deporte e Informática, Facultad del Deporte, Universidad Pablo de Olavide, Sevilla, Spain; Instituto de Química - Universidade de São Paulo, Brazil

## Abstract

Every other day feeding (EOD) and exercise induce changes in cell metabolism. The aim of the present work was to know if both EOD and exercise produce similar effects on physical capacity, studying their physiological, biochemical and metabolic effects on muscle. Male OF-1 mice were fed either *ad libitum* (AL) or under EOD. After 18 weeks under EOD, animals were also trained by using a treadmill for another 6 weeks and then analyzed for physical activity. Both, EOD and endurance exercise increased the resistance of animals to extenuating activity and improved motor coordination. Among the groups that showed the highest performance, AL and EOD trained animals, ALT and EODT respectively, only the EODT group was able to increase glucose and triglycerides levels in plasma after extenuating exercise. No high effects on mitochondrial respiratory chain activities or protein levels neither on coenzyme Q levels were found in gastrocnemius muscle. However, exercise and EOD did increase β-oxidation activity in this muscle accompanied by increased CD36 levels in animals fed under EOD and by changes in shape and localization of mitochondria in muscle fibers. Furthermore, EOD and training decreased muscle damage after strenuous exercise. EOD also reduced the levels of lipid peroxidation in muscle. Our results indicate that EOD improves muscle performance and resistance by increasing lipid catabolism in muscle mitochondria at the same time that prevents lipid peroxidation and muscle damage.

## Introduction

Every other day feeding (EOD) is a procedure that resembles many of the effects of caloric restriction (CR). EOD increases life-span, decrease cancer incidence and protects against age-associated diseases and neurodegeneration or endogenous damage of DNA [1]–[4]. EOD is considered to produce a mild CR inducing around a 15% of reduction of the total ingestion of calories. On the other hand, exercise increases energy expenditure and affect morbidity and lifespan in a similar way than CR on morbidity and lifespan [5], [6]. Regular physical activity induces analogous cellular and molecular changes in cardiovascular and nervous systems to those observed in animals fed under CR conditions [4]. Furthermore, endurance exercise and CR delay the progression of immunosenescence [7] and induce a general anti-inflammatory effect preventing the age-dependent increase of plasmatic C-reactive protein levels in rats [8].

The similar effects induced by CR and exercise suggest that both interventions share some mechanisms that modify cell physiology. *Ex vivo* experiments on rat gastrocnemius muscle exposed to electrical stimulation have suggested synergy between CR and aerobic exercise in muscle bioenergetics [9]. In humans, the practice of aerobic exercise together with CR improves insulin sensitivity and reduces plasmatic LDL levels [10] and also seems to improve neurocognitive function [11]. However, in other cases, CR and exercise induce contrary effects such as in the decrease of diet-induced weight loss muscle mass in cases of intentional weight loss in old individuals [12].

Both, CR and exercise modify significantly the bioenergetics of muscle. Long-term CR delays the decline of the skeletal muscle aerobic capacity that occurs during aging [13]. Endurance exercise and CR promote changes in muscle fibers and mitochondrial activity by increasing the activity of PPAR-δ [14]–[16]. Interestingly, during aging, the activity of complexes involved in aerobic energy production decreases in muscle whereas both CR and exercise maintain them [17]. It is known that CR induces changes in mitochondrial biogenesis and activity through activation of SIRT-1 and PGC1α-dependent mechanisms [18]. Moreover, resveratrol, a known polyphenol considered a mimetic of CR, also induces changes in muscle mitochondrial activity and improves physical performance in mice [19], [20] and could be considered as an ergogenic factor able to modify muscle physiology [21].

Most of the studies reported about dietary restriction and exercise have been performed by comparing the effect of CR versus exercise [22], [23]. There are few data available about the effect combined effect of CR and exercise in metabolism and muscle performance. Thus, the aim of this work is to study the effect of the combination of endurance training and EOD on physiological, molecular and metabolic aspects affecting muscle activity. We hypothesize that some of the modifications induced by EOD mimic the effect of aerobic exercise and improve physical performance. It is possible that EOD and exercise could show additive effects in some capacities. In fact, the results shown here suggest that EOD by itself or together with moderate physical activity positively affects muscle performance by increasing the metabolism of lipids by β-oxidation of fatty acids, increasing fuel mobilization during exercise and preventing oxidative stress and also muscle damage.

## Results

### Effects of EOD and training on physical activity

**Figure 1:**
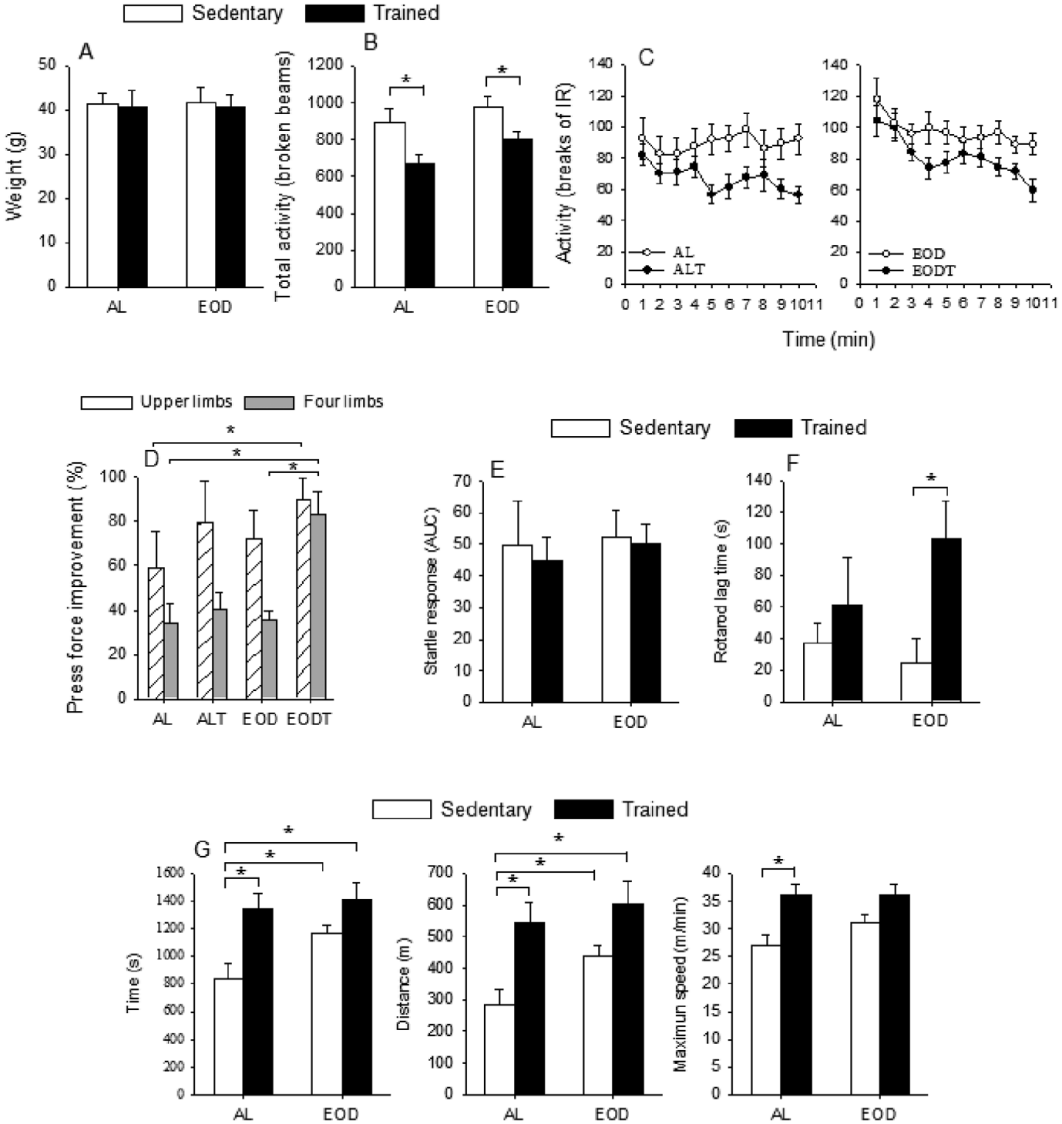
Physical performance analysis. A) Weight of animals at the end of the experiment (n = 16). B) Exploratory locomotion activity in broken beans after 10 min. C) Time course of periphery exploration of animals (n = 16). D) Grip strength of upper limbs (left) and four limbs (right). E) Startle response measured as area under curve. F) Rotarod lag time at 100 rpm. G) Time after exhaustion on treadmill (left), distance (middle) and maximum speed reached (right). X axis in each figure indicates the groups AL or EOD. In figures A, B and E–G open bars indicate sedentary animals whereas closed bars indicate trained animals in each group. * Significant differences *vs.* indicated group, p≤0.05; ** Significant differences *vs.* indicated group, p≤0.005.

The scope of our research includes the study of different physical activities such as: spontaneous locomotion, grip strength, startle response, motor coordination and resistance to strenuous exercise in mice submitted EOD and/or endurance exercise. All these parameters might be influenced by difference in mice's weight since they were fed in different conditions. However along our experiment we did not find any difference in weight between groups under either AL or EOD (Fig. 1A). Furthermore, endurance exercise did not produce any significant modification on the weight of animals. Determination of animal's locomotion by measuring the movement of each animal on an open field did not show differences between the sedentary groups (Figs. 1B–C). However, training significantly decreased both free activity of animals and peripheral exploration probably by a higher habituation capacity of these animals.

To determine the effect of EOD and/or exercise on muscle capacity we determined grip strength, startle assay, rotarod coordination and strenuous exercise tests. Grip strength was used to determine the improvement for the press force after the 6 weeks of training. We found that exercise significantly improved press force in four limbs (86.88±12.17% of improvement in ALT+EODT animals, n = 32, *vs.* 66.05±10.41% in AL+EOD animals, n = 32, *P* = 0.002). Further, EOD also produced significant improvement in this test (82.77±10.51% in EOD+EODT animals, n = 32, *vs.* 69.93±12.49% in AL+ALT animals, n = 32, *P* = 0.019). When we compared the four groups, the higher and more significant improvement was found in the EODT group (Fig 1D) affecting both two and four limbs tests. Muscle potency, determined by using the startle assay showed no differences between groups (Fig. 1E). Motor coordination and resistance was determined carrying out a specific test designed by using a Rotarod. Animals were forced to resist onto an accelerating Rotarod until 100 rpm, once reached this speed, the time each animal remained on the device was quantified. Training increased motor coordination being significantly higher in EODT animals in comparison with sedentary EOD animals (*P* = 0.001), (Fig. 1F).

Finally, we carried out an extenuating endurance test. Eight animals of each group ran until extenuation. Among all the groups, EODT was the group that ran longer and covered more distance (Fig. 1G). Among non-trained animals, EOD group showed a significant higher resistance in comparison with AL group (1167±57 *vs.* 840±108 s, *P* = 0.015) and covered more distance (603.7±69.4 *vs.* 285.6±47.6 m, *P* = 0.003). Remarkably, EOD group showed almost the same capacity than trained groups (Fig. 1G), indicating that EOD itself was able to increase muscle resistance mimicking endurance exercise training.

### EOD and training affect nutrients mobilization during exercise

**Figure 2:**
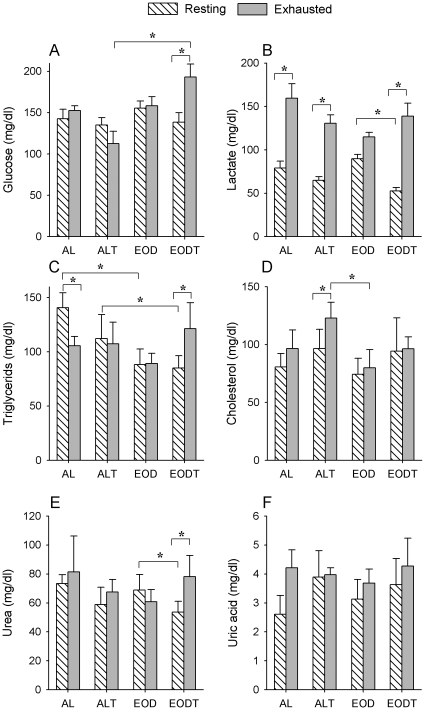
Metabolites in plasma of animals. Levels of metabolites in plasma of witness animals are indicated in white and plasma levels of animals just after strenuous exercises are indicated in black. A) Glucose, B) Lactate, C) Triglycerides, D) Cholesterol, E) Urea, F) Uric acid. * Significant differences vs. indicated group, p≤0.005.

The two EOD groups were able to maintain or even increase (in the case of EODT) glucose levels after extenuating exercise (Fig. 2). A mean of 37% increase of glucose level was found in the EODT group after extenuating exercise (*P* = 0.014). In contrast, plasma glucose levels decreased in the ALT group after exercise although showing similar performance in the extenuating test (Fig. 2A). In the AL group, glucose levels did not changed but we have to remark that the resistance of this group was considerably less than in both EOD groups. In fact, when we analyzed the levels of glucose of both EOD groups in comparison with AL groups, a significant increase of glucose in plasma after extenuating exercise was found (140.0±11.8 mg/dl in AL+ALT animals, n = 32, *vs.* 185.4±14.2 in EOD+EODT animals, n = 32) (*P* = 0.012).

In the case of lactate, levels of witness animals showed a tendency to decrease in trained animals (Fig. 2B) and especially in the EODT group. After exercise, all the groups, except EOD, showed a significant increase in plasmatic lactate, even in the AL group besides the poor performance.

**Figure 3:**
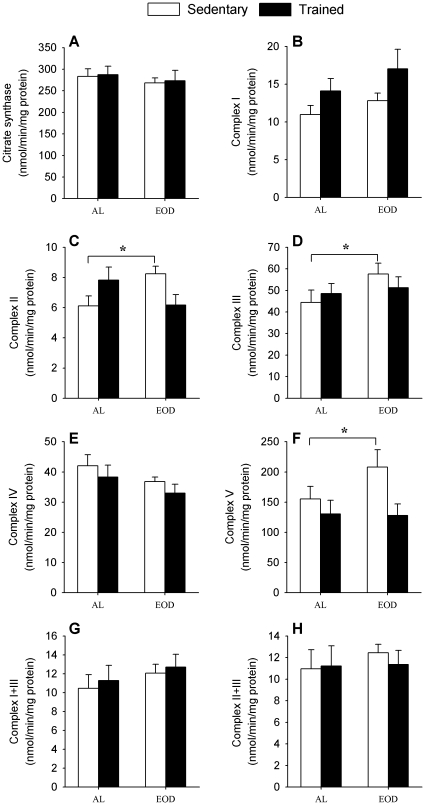
Mitochondrial activities in whole muscle homogenate. Indicated activities were determined as nmol/min/mg protein. A) Citrate synthase, B) Complex I (NADH:ubiquinone oxidoreductase), C) Complex II (succinate dehydrogenase), D) Complex III (ubiquinol:cytochrome c oxidoreductase), E) Complex IV (cytochrome c oxidase), F) Complex V (ATP synthase), G) Complex I+III (NADH:cytochrome c oxidoreductase), H) Complex II+III (succinate:cytochrome c oxidoreductase). * Significant differences vs. indicated group, p≤0.05.

In the case of lipids, levels of circulating triglycerides (TGs) in witness animals were significantly lower in animals fed under EOD conditions (86.7±3.4 mg/dl in EOD+EODT animals, n = 32) *vs.* AL animals (125.3±6.7 mg/dl in AL+ALT animals, n = 32) independently of training (*P* = 0.001). After extenuating exercise, EOD and EODT groups were able to maintain or even increase TGs levels while they decreased in AL group (Fig. 2C). As in the case of glucose, the EODT group showed an increase of around 42.5% in circulating TGs after exercise. In the case of cholesterol, training induced higher plasmatic levels before extenuating exercise independently of nutrition (97.1±4.1 mg/dl in ALT+EODT animals, n = 32 *vs.* 80.0±5.2 mg/dl in AL+EOD animals, n = 32, *P* = 0.02). After extenuating exercise, both AL and ALT groups showed increases in plasmatic cholesterol while EOD and EODT groups did not present any changes respecting witness animals (Fig. 3D). This increase was significantly different (*P* = 0.017) when all AL fed animals (AL+ALT: 111.0±6.0 mg/dl, n = 32) were compared with all animals fed under EOD conditions (EOD+EODT: 89.2±5.9 mg/dl, n = 32).

Finally, in the case of urea lower basal levels were found in trained animals (ALT+EODT: 56.3±2.7 mg/dl, n = 32) respecting to sedentary animals (AL+EOD: 73.2±5.7 mg/dl, n = 32) (*P* = 0.011). The effect of exercise on initial levels of urea was higher in EOD groups being significant between EOD and EODT groups (*P* = 0.042). Extenuating exercise did not produce any remarkable change in the levels of plasmatic urea except in the case of EODT group (Fig. 3E). In the case of uric acid, no significant differences of levels between groups were found although we did find a tendency to increase after extenuating exercise. Similar small modifications were also found in the levels of albumin in plasma (data not shown).

### EOD and training modify in different degree mitochondrial muscle activities

A higher physical resistance can be explained by changes in muscle metabolism. Thus, we proceeded to determine mitochondrial activities in gastrocnemius muscle (Fig. 3). In the case of citrate synthase (CS) activity, a marker of mitochondrial mass, we did not find any substantial differences in whole muscle homogenates between mice groups in CS activity (Fig. 3A).

Mitochondrial function was determined by quantification of respiratory complexes activities. Training and EOD induced a slight although not significant increase of complex I activity (Fig. 3B). Complexes II and III activity of the EOD group increased significantly respect to AL group (*P* = 0.014 and *P* = 0.045 respectively). On the other hand, training slightly but not significantly increased activity in AL fed animals (*P* = 0.148, ALT vs. AL in complex II and *P* = 0.868 in complex III). However, in EODT animals a decrease of activity vs. EOD animals was found being significant in the case of complex II (*P* = 0.017) but not in the case of complex III (*P* = 0.379) (Figs. 3C–D). No changes were found in complex IV (Fig. 3E). In the case of the activity of complex V (Fig. 3F) we found a non-significant increase in EOD group when compared with AL group (*P* = 0.183). Training induced a slight decreased of this activity in AL fed animals (*P* = 0.402) being higher and significant in EOD fed animals (*P* = 0.035). We also determined the coupled activity of complexes I+III and II+III that depend on coenzyme Q (Q) amount in mitochondrial membrane. No changes among groups were found in both activities (Figs. 3G–H).

**Figure 4:**
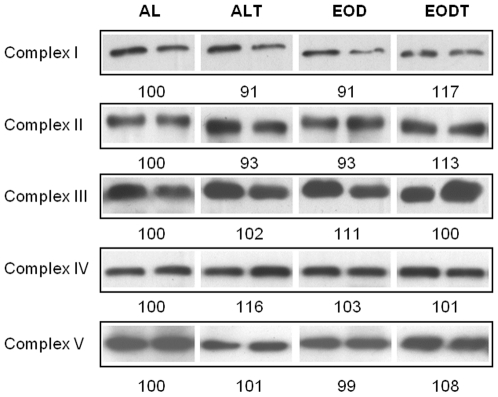
Levels of proteins markers of each mitochondrial complex. Proteins from whole homogenate were resolved by SDS-PAGE electrophoresis and the presence of the markers for each complex was determined by immunoblotting as indicated in Material and Methods section. Blots were quantified by densitometry and normalized vs. protein loading determined by membrane staining with red Ponceau. Numbers indicate the mean (n = 5) in arbitrary units normalized vs. AL group as 100. No significant differences were found between groups.

When we analyzed the amount of protein complexes of mitochondria no significant changes among groups were found in any of the four respiratory complexes and ATPase (Fig. 4). Then, our results suggest that the small changes found in the activity of mitochondrial complexes may be due to post-translational changes. We also proceeded to quantify Q in whole muscle homogenate. Q_9_ is the predominant form in mice being around 10 times more abundant than Q_10_. No significant differences among the different mice groups were found (Table 1). However, EOD animals showed a non-significant increase of the amount of Q_9_ and total Q in comparison with AL group similarly to the effect found with training. On the other hand, Q_10_ increases in trained animals but not in dietary restricted animals. This different effect affected the ratio between Q_9_ and Q_10_ that increased in EOD groups whereas decreased in trained animals.

**Table 1:** Coenzyme Q levels in gastrocnemius muscle.

	Q_9_	Q_10_	Q total	Ratio Q_9_/Q_10_
**AL**	1245±154	122±19	1367±171	10.8±0.7
**ALT**	1298±121	139±18	1384±150	9.2±0.5
**EOD**	1352±65	116±5	1464±69	11.8±0.5
**EODT**	1293±123	127±16	1420±139	10.9±0.6

Data represent the average (pmol/mg protein from whole homogenate) ± SE, n = 16.

**Figure 5:**
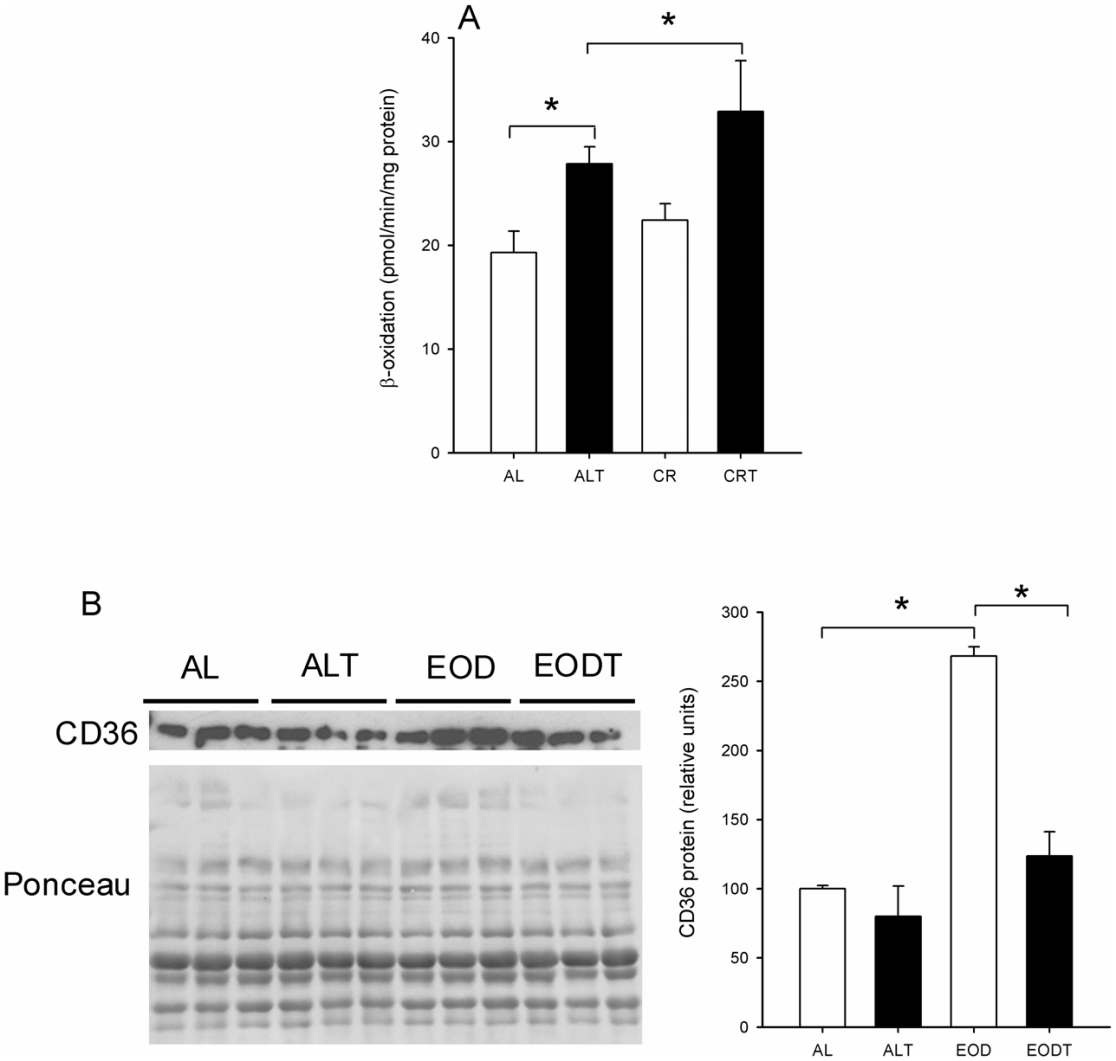
Lipid catabolism in muscle. A) β-oxidation in whole muscle homogenate (pmol/min/mg protein). B) Levels of CD36 measured from whole muscle homogenate. Left, CD36 levels measured by immunoblotting and respective Ponceau staining of proteins transferred to membrane. Right, densitometry analysis of CD36 protein levels/protein loading. * Significant differences vs. indicated group, p≤0.05.

We also analyzed the activity of β-oxidation in muscle (Fig. 5A). Training significantly increased β-oxidation activity in gastrocnemius muscle (*P* = 0.050), whereas EOD only induced a non-significant increase (*P* = 0.305). However, when combined, EOD and exercise highly increased β-oxidation in comparison with the levels found in the AL group (*P* = 0.027). Further, we determined the level of the fatty acid translocase (CD36), a protein involved in the incorporation of fatty acids from plasma to muscle and in mitochondrial fatty acid oxidation. Surprisingly, EOD significantly increased CD36 levels in comparison with AL animals (*P* = 0.0009) whereas in EODT animals the levels of this protein decreased to the levels of AL group (Fig. 5B).

**Figure 6:**
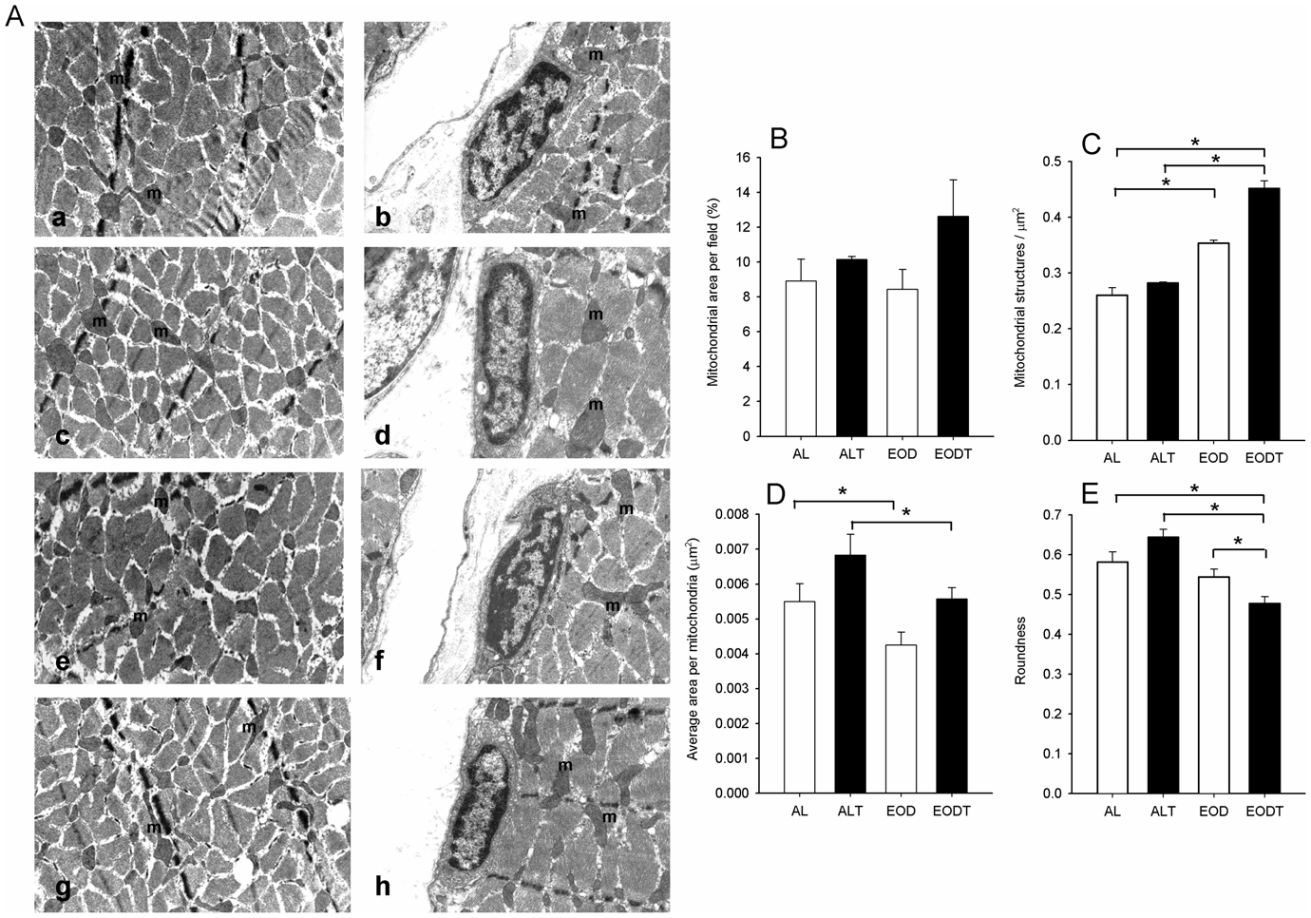
Mitochondrial structures in gastrocnemius muscle. A) Electron micrographs of gastrocnemius muscle. Left (a,c,e,g), ×5,200; right (b,d,f,h), a higher detail of mitochondria in gastrocnemius muscle and nucleus of satellite cell, ×8,900; a,b: ad libitum (AL) group, c,d: trained ad libitum group (ALT), e,f: every-other day fed group (EOD), g,h: trained every-other day fed group (EODT); m: indicates the presence of mitochondria. B) Mitochondrial area per field in muscle from each group (n = 10 per group). C) Number of mitochondrial structures per µm2 (n = 10 per group). D) Average area per mitochondria (n>100). E) Roundness of mitochondria (n>100). Error bars indicate SE. * Indicates significant difference vs. indicated group p≤0.05.

Electron micrographs of gastrocnemius muscle were also analyzed (Fig. 6A). Quantification of intermyofibrillar mitochondria in muscle demonstrated that, in general, the surface occupied by mitochondria did not show significant differences between the four groups (Fig. 6B). However, when we determined the number of mitochondrial structures per surface unit, we found that EOD induced a significant increase being higher in the case of the EODT group (Fig. 6C). Training increased the average area per mitochondria in both, AL and EOD animals, whereas EOD induced a clear decrease in this area (Fig. 6D). Differences in shape were determined by measuring the roundness of mitochondria. EOD significantly decreased this parameter indicating more longitudinal mitochondria (Fig. 6E). These parameters confirmed the observations of micrographs indicating that training induced hypertrophy by increasing the size of mitochondria in muscle fibers whereas EOD modified their shape and localization increasing the amount of intermyofibrillar mitochondria and modifying the shape of these mitochondria that appeared longer and sinusoid around the myofibrils.

### EOD prevents exercise-induced muscle damage

**Figure 7:**
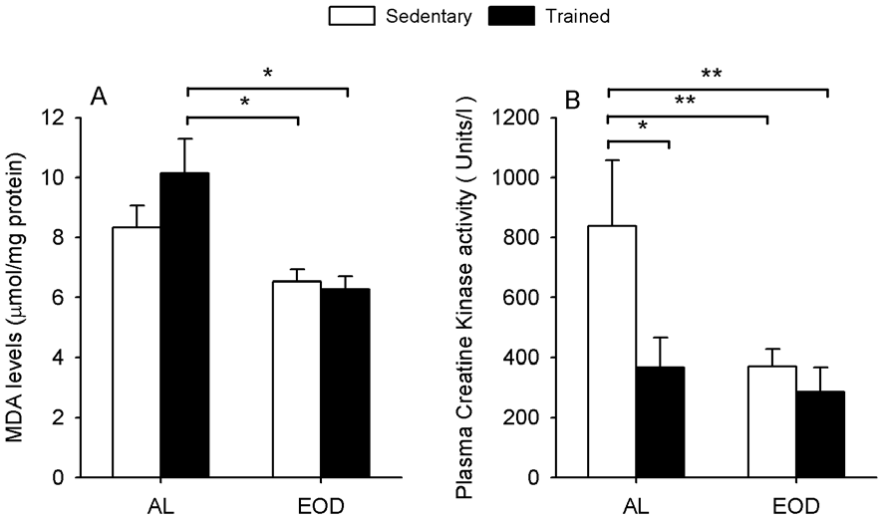
Levels of damage in muscle. A) Levels of the marker of oxidative damage MDA in whole muscle membranes. B) Plasma creatine kinase activity as marker of muscle damage after strenuous activity. * Significant differences vs. indicated group, p≤0.05; ** Significant differences vs. indicated group, p≤0.005.

Accumulation of oxidative damage can be one of the causes of muscle fatigue. For this reason we determined the levels of lipid peroxidation in gastrocnemius muscle. No differences were found between witness and extenuated animals probably by the short time passed between extenuation and sacrifice. Analysis of the lipid peroxidation in the whole population of each group indicated that ALT animals showed the highest levels of MDA (Fig. 7). On the other hand, both groups of EOD animals showed significant lower levels of MDA indicating that EOD protected muscle against lipid peroxidation in muscle independently of training.

Levels of muscle damage after extenuating exercise were also determined by the presence of creatine kinase activity in plasma. Besides the lower physical resistance, AL animals showed the higher muscle damage of all groups (Fig. 7). On the other hand, non-trained EOD animals showed similar levels of muscle damage than trained animals (ALT and EODT) indicating a similar degree of resistance of muscle to damage.

## Discussion

Many studies about CR are based on a decrease of 40–50% calories in the daily uptake of food. In our study we have used the every-other day feeding model (EOD), which is considered to produce only a mean of 15% deficit in the calorie input due to the fact that animals eat more when they have access to food [24]. Although EOD did not produce a high caloric deficit, it has been demonstrated that EOD mimics many of the beneficial aspects of classical CR. However, the effect of this model on longevity is currently under discussion [25] including higher longevity and higher resistance to stress [1], [4], [26]–[29]. The advantage of the EOD model in our study is that it did not affect the weight of animals during the study and thus, weight, size and nutrients availability were not factors that could influence the physical capacity or muscle performance of the animals.

Edge exploration and elevated plus maze tests indicate that the differences in physical activity were not due to a different degree of anxiety between groups. However, an interesting finding was that, with independence of the feeding procedure, trained animals decreased their total activity in an open field after few minutes probably indicating a higher capacity to recognize new environments. Our outcomes have also shown that EOD and aerobic training increase motor coordination and resistance to exhausting exercise. In the case of running on rolling carpet, EOD slightly increased physical resistance in trained animals whereas it did significantly increase resistance in the sedentary group when compared with the AL group. Interestingly, one of the documented changes in rodents fed under CR is the increase of non-forced physical activity when compared with AL fed animals [30], [31].

Another interesting issue was the effect of EOD on the capacity of mobilization of nutrients such as sugars and fats. Our study differs from others mainly in the fact that metabolite analysis of blood was performed in animals that were not fasted overnight. Our aim was to determine the physical capacity and associated parameters, thus, we could not submit the animals to strenuous exercise after fasting. Our data indicate that EOD improves the capacity of mobilization of glucose and lipids maintaining the levels of these metabolites during a strenuous exercise. These results suggest that animals under EOD were more efficient in mobilizing nutrients necessary for physical activity. In agreement with our results, a study made on body builders has proved that restriction in calories during a brief period of time (7 days) increases the capacity to mobilize lipids during running [32].

Induction of mitochondrial biogenesis by CR or exercise has been shown in different organisms and tissues [33]–[35]. We already demonstrated that CR increases mitochondrial biogenesis and modifies activity of mitochondria in liver and cell culture models [36]. However, in the present model of CR we have not found changes in mitochondrial mass determined by CS activity neither notable changes in mitochondrial respiratory chain proteins. This is not due to the EOD model of CR used in our project since, in agreement with our results, no significant changes in CS activity have been also reported in rats fed under classical CR [37]. Further, it has been suggested that only two weeks of CR are able to significantly decrease CS activity and other mitochondrial activities in whole muscle homogenate of Sprague Dawley rats [38]. Furthermore, 40% CR reduces CS activity in rat gastrocnemius muscle [39]. CR also induces the decrease of the activities of complexes I, III and IV especially in young mice [40].

The increase in mitochondrial content is considered a well-established adaptation of muscle to exercise, and is mainly referred as mitochondrial biogenesis. In our hands, neither EOD nor exercise induced changes in the activity and amount of proteins involved in respiratory chain. Similar results have been recently shown in other models of CR and exercise where no changes in porin, a mitochondrial mass indicator, have been reported [41]. In agreement with our results, it has been reported that similar protocols of endurance exercise does not produce changes in β-oxidation in gastrocnemius muscle of mice [42] neither in other activities of the respiratory chain in rat gastrocnemius muscle [43]. These discrepancies can be explained because in most cases mitochondrial biogenesis in muscle is only suggested by the increase of PGC1α mRNA levels. However, recently Civitarese et al. [44], have described that the expression of genes encoding proteins involved in muscle mitochondrial biogenesis is induced by CR and CR plus exercise in healthy humans but at the same time neither CS activity nor β-oxidation and activity of electron transport chain are affected. Furthermore, no effects of CR on mitochondrial activity have been reported in rats besides the increase of the transcription of genes involved in energy metabolism [37]. These results indicate that induction of mitochondrial biogenesis factors are not necessarily accompanied by significant changes in mitochondrial mass or activities. In our experiments, EOD did induced small significant changes in the activity of mitochondrial complexes II and III indicating that the effect of EOD on mitochondrial activity may be due to modifications of the activity of mitochondrial complexes but not by changes in the amount of these complexes. We have previously shown that CR improves the efficiency of mitochondria in cells [36]. In agreement with this suggestion, other reports have suggested that CR preserves the oxidative capacity of muscle by protecting mitochondrial function rather than content [39]. Taken together, small and fine modifications of mitochondrial activities, rather than significant increases of mitochondrial mass, may be enough to maintain a more equilibrated and effective mitochondrial activity in muscle and to enhance their efficiency.

Our results also indicate that both EOD and exercise increase β-oxidation in muscle. Electron microscopy analysis of the mitochondria in gastrocnemius muscle demonstrated that the size, localization and morphology of mitochondria were different in EOD animals. EOD and EODT animals showed more mitochondrial structures in the intermyofibrillar area and around the myofibers. Intermyofibrillar location of mitochondria has been associated with lipid droplets. Thus, these changes in the mitochondria in an intermyofibrillar location in muscle suggest a more efficient activity producing ATP near the sarcomera by using lipid catabolism [45]–[47]. In agreement with our results, recent works have suggested that changes in mitochondrial morphology affect intracellular energy levels and also mitochondrial activity [48], [49]. Furthermore, the levels of the fatty acid translocase (CD36) increased in EOD group indicating a higher capacity to import lipids from plasma and also a higher mitochondrial fatty acid metabolism [50], [51]. Further, higher amounts of glycogen and TGs have been also found in muscles of rats after EOD conditions [52] indicating a higher capacity to use internal fuel sources during exercise.

In agreement with previous results, levels of Q were also slightly affected by EOD and exercise [53]. Very interestingly, EOD and training seem to modify the ratio between Q_9_ and Q_10_ in a different way. Exercise decreased the ratio whereas EOD increased it. These changes probably respond to a more bioenergetic function of Q_9_ whereas Q_10_ mainly plays an antioxidant role in mice. CR decreases the levels of reactive oxygen species (ROS) in cells [36] and increases Q-dependent reductases activity protecting cell membranes against oxidative damage [54], [55]. On the other hand, depending on the intensity, exercise increases free radical production and induces oxidative stress [56], [57]. Therefore, under CR conditions, the role of Q must be displaced to benefit bioenergetics instead of antioxidant aspects that are covered by the increase of cellular antioxidant capacity of the cell and then, Q_9_/Q_10_ ratio increases [54], [55]. On the other hand, the increase of ROS induced by exercise will need more Q-dependent antioxidant protection and then, the ratio decreases. It seems that the equilibrium between Q levels and Q-dependent activities must be important to maintain a balanced muscle activity [58].

EOD protected muscle fibers against oxidative stress even in animals that suffered endurance activity. This higher protection may be related to lower muscle damage after extenuating activity in non-trained EOD animals determined by the levels of creatine kinase in plasma. Our results agree with several other reports that suggest that EOD protects muscle against oxidative stress and even avoids muscle loss during aging [59], [60]. This protection is based on lower ROS production by mitochondria probably by the metabolic changes based on the catabolism of lipids [61], by more balanced activity of mitochondria [36] and higher level of Q-dependent antioxidant protection of membranes [55].

In summary, our work indicate that a nutritional stress induced by the EOD model of CR together with a moderate increase of energy expenditure through physical exercise produces metabolic changes that increase the efficiency of mitochondrial activity in muscle, reduces oxidative damage and improves physical performance. Subtle modifications at the cellular and biochemical levels in response to dietary stress seem to be the basis for a higher mitochondrial efficiency. Taking into consideration that the decline in physical activity during age is a common factor in many species, the results shown here suggest that the combination of the reduction of calorie intake and the practice of aerobic exercise would also increase physical performance in humans and then, improve their quality of life.

## Materials and Methods

### Animals

A cohort of 64 non-consanguineous swiss-OF1 male mice aged 6 weeks was used (Animal Services, University of Granada, Granada, Spain). Animals were housed into enriched environmental conditions in groups of 6 animals per polycarbonate cage in a colony room under a 12 h light/dark cycle (8:00 AM–8:00 PM) under temperature (22±3°C) and humidity controlled. Animals were maintained accordingly to a protocol approved by the Pablo de Olavide University Ethical Committee and following the international rules for animal research.

### Caloric restriction and endurance training

Animals were first randomly assigned to two initial groups: half of the animals were fed *ad libitum* (AL) and the other half under CR using the every-other-day feeding model (EOD) [1]. Water was available *ad libitum* for all the groups. After four months under these conditions, each group was randomly subdivided again in a sedentary group and a trained group following a mild forced aerobic exercise protocol. During the first two weeks, a training protocol was performed by a routine increasing both speed and time on a treadmill (Treadmill Columbus 1055M-E50, Cibertec SA) until reaching 20 meters/min and 20 min. This protocol consisted in the 70–80% of average maximum speed reached in the initial tests. After that, animals were trained at this speed for 20 min/5 days a week during the following 6 weeks similarly to already published endurance exercise protocols [42]. The final animals groups were as follows: AL, *ad libitum* and sedentary group; ALT, *ad libitum* and trained group; EOD, caloric restricted and sedentary group and EODT, caloric restricted and trained group. Weight was determined every 15 days.

### Physical and behavioral activity analysis

All physical activity analysis was carried out between 9–11 hours in the morning and just after a feeding day for the EOD groups. Spontaneous activity was determined by using an open field box (26×39 cm) (Cibertec S.A., Madrid, Spain) determined by the number of broken light beams during a period of 10 min per animal. Peripheral exploration was determined directly by eye in sessions of 5 min as anxiety measure. Also, elevated plus maze test was used to quantify anxiety levels during 5 min per animal. Press force tests were performed by using a Grip Strength (Columbus, Cibertec SA, Spain). For startle response, animals were placed individually inside a startle chamber (Cibertec SA, Spain). The startle response was determined by using a piezoelectric accelerometer controlled by homemade software. Startle stimulus was 100 ms at 125 db and the response is indicated as the average from 20 to 30 recordings. Motor activity and coordination tests were performed on Rotarod (Hugo Bassile, Italy). After an adaptation period, a test was performed to combine coordination and resistance by using Rotarod speed acceleration up to 100 rpm and determining the maximum riding time of animals at this speed.

In the case of extenuating activity, all groups of animals performed a week of habituation before test. Half of the animals from each group (n = 8) were exposed to extenuating physical exercise on treadmill associated with electric stimuli, without inclination and fastening speed by 5 meters/min every 5 min. We established the end of the experiment at the moment the animal stopped for more than 5 seconds under electric stimuli without trying to move back to the treadmill. Animals were sacrificed by cervical dislocation and dissection was performed just after the test was finished. The rest of the animals of each group remained as witnesses without running and were sacrificed at the same time than the runners. These witness animals were considered as controls of the plasmatic metabolic situation of animals before exercise. To avoid any effect of nutrient uptake on extenuating activity performance, all groups received food during the day before the exercise test.

### Analysis of plasmatic compounds

Blood was collected by cardiac puncture just after cervical dislocation. Plasma was obtained by centrifugation in Vacuette Z serum Sep. Clot activator tubes for 10 min at 3000×*g* and stored in small aliquots kept at −80°C until the determination of different blood metabolites. Metabolites were analyzed by using commercial kits for triglycerides (Randox TR210), cholesterol (Randox CH201), urea (Randox UR457), uric acid (Randox UA233), L-lactate (Randox, LC2389), glucose (Randox GL2623), total protein (Randox TP245) and albumin (Randox, AB388). Creatine kinase (CK) activity was analyzed by the CK-NAC (Randox, CK113) test. In all cases, samples were always processed in parallel with the respective quality controls provided by the supplier.

### Muscle mitochondrial activities

Gastrocnemius muscles were dissected immediately after sacrifice and frozen in liquid nitrogen. After thawing and clearing from connective tissue, muscle was homogenized by using lysis buffer (2 mM Tris-HCl, 20 mM Hepes, 1 mM EDTA, 70 mM sucrose and 220 mM Mannitol) supplemented with 1 mM PMSF and protease inhibitor cocktail 1∶50 (Sigma) in a 1∶9 volume-to-weight ratio, followed by centrifugation for 10 min at 700×*g* to eliminate debris and nuclei. Protein was determined by Stoschek-modified Bradford's method [62]. Activities are indicated as nmol/min/mg protein.

Mitochondria chain complexes activities were measured in a spectrophotometer (Thermo Spectronic Unicam UV 500). Complex I was measured in 20 mM phosphate buffer pH 8.0 by kinetic quantification of 0.2 mM NADH consumption at 340 nm for 2–3 min at 30°C after adding 1 mM CoQ_1_ as electron acceptor (ε_340_: 6.81 mM^−1^ cm^−1^). Complex II was measured in 50 mM phosphate buffer pH 7.0 by kinetic quantification of the reduction of 0.1 mM diclorophenol-indophenol (DCPIP) at 600 nm for 2 min at 30°C after adding 32 mM succinic acid to the reaction (ε_600_: 19 mM^−1^ cm^−1^). Complex III was determined in 50 mM phosphate buffer pH 7.5 by kinetic quantification of the reduction of the cytochrome C (0.05 mM) at 550 nm for 2 min at 30°C after adding decylubiquinol (0.05 mM) as electron supplier to the reaction (ε_550_: 21 mM^−1^ cm^−1^). Complex IV was measured in 10 mM phosphate buffer pH 7.0 by kinetic quantification of oxidation of reduced cytochrome C as electron donor at 550 nm for 2 min at 38°C using oxygen as electron acceptor (ε_550_: 21 mM^−1^ cm^−1^). The combined activities of complex I+III and II+III were determined in 50 mM phosphate buffer pH 7.5 by determining the kinetics of the reduction of cytochrome C for 2 min at 30°C by adding NADH (0.1 mM) (complex I+III) or succinic acid (3 mM) (complex II+III) as electrons donors (ε_550_: 21 mM^−1^ cm^−1^). For each determination, specific inhibitors such as rotenone (Complex I, 5 µM), actimycin A (Complex III, 1 µg/ml), sodium azide (Complex IV, 1–2 mM) and ferric cyanide (Complex IV, 1.5 mM), were used as previously indicated [63].

Complex V/ATPase activity was determined by the measurement of its ATP phosphatase activity. Assay was performed in Hepes-Mg pH 8.0 (50 mM) by adding NADH (0.2 mM), phosphoenol pyruvate (2.5 mM), pyruvate kinase (10 mg/ml), lactate dehydrogenase (5 mg/ml) and antimycin A (0.2 mg/ml). After 2 min of incubation, ATP (25 mM) pH 7.0 was added (ε_340_: 6.22 mM^−1^ cm^−1^). Inhibition with oligomycin (0.2 mg/ml) was used to specifically determine mitochondrial ATPase activity.

Citrate synthase (CS) activity was determined in 75 mM Tris-HCl buffer (pH 8.0) by quantification of ditio-bis-nitrobenzoate (0.1 mM) reduction in the presence of Triton X-100 1%, acetyl CoA (7 mg/ml) and oxalacetate (5 mM) at 412 nm for 2 min at 30°C (ε_412_: 13.6 mM^−1^ cm^−1^).

### Measurement of β-oxidation Activity

Palmitate oxidation was measured as an indicator of β-oxidation [64]. Briefly, the reaction mixture contained 50 ml Tris/HCl, pH 8.0, 40 mM NaCl, 2 mM KCl, 2 mM MgCl_2_, 1 mM DTT, 5 mM ATP, 0.2 mM L-carnitine, 0.2 mM NAD, 0.6 mM FAD, 0.12 mM CoA, 0.1 µCi [^14^C]palmitic acid, and 100 µg of whole muscle homogenate in a final volume of 200 µl. The reaction was initiated by adding substrate and incubated at 37°C for 60 min. Reaction was terminated by adding 200 µl of 0.6 N perchloric acid followed by centrifugation. The resulting supernatant was extracted three times with 800 µl of N-hexane to remove any remaining palmitate and radioactivity of the aqueous phase was measured.

### Coenzyme Q determination

One to 1.5 mg of protein of whole muscle homogenates were resuspended in 500 µl of PBS 1x and incubated with SDS (1% final concentration) for 10 min followed by a vigorous shake with vortex. Q was extracted with hexane as indicated [63]. Briefly, 2 volumes of ethanol:isopropyl alcohol (95∶5) were added to SDS solution and mixed with vortex for 1 minute. The organic phase was recovered by the addition of 4 volumes of hexane, shaking for 1 min in vortex and centrifugation at 1,000×*g* for 5 min at 4°C. The higher phase containing Q was recovered and extraction was repeated. Hexane phase was dried by using a Rotavapor and residue resuspended in 1 ml of ethanol HPLC grade. Ethanol was dried again by using a Speed Vac and residue was kept at −20°C until determination. After dissolving again in ethanol, Q levels were determined by using a HPLC equipped with a Spherisorb C-18 column at a flow of 1 ml/min and a UV/Vis Beckman detector and an Electrochemical ESA Coulochem III. Q_6_ and decylubiquinone were used as internal controls. Q concentrations are indicated as pmol/mg protein.

### Western blotting

Whole muscle homogenate was obtained after disruption in lysis buffer as above indicated by using a mechanical driven homogenizer and further centrifugation at 700×g to remove debris. Supernatant was mixed with two times concentrated Laemmli Buffer (Santa Cruz Biotechnology, USA) and proteins separated by SDS-PAGE. Proteins were transferred to Hybond ECL nitrocelullose membranes (GE-Healthcare, USA) and visualized by using the mouse monoclonal antibodies against the 39 kDa subunit of NADH:ubiquinone oxidoreductase (clone 20C11, complex I), 70 kDa subunit of Succinate:ubiquinone oxidoreductase (clone 2E3, complex II), core 2 subunit Ubiquinol:cytochrome c oxidoreductase (clone 13G12, complex III), subunit Vb of the Cytochrome c oxidase (clone 16H12, Complex IV) and α-subunit of ATP synthase (clone 7H10, complex V) (Invitrogen-Molecular Probes, USA) at 1∶1000 dilution and HRP-labeled anti-mouse IgG at 1∶1000. Blots were visualized by ECL technique and quantified by using the Quantity One 1-D analysis software (Biorad). Blots were digitalized and quantified by using Quantity One software (Biorad). Protein expression levels were corrected for whole protein loading determined by staining membrane with Red Ponceau.

### Measurement of Malondialdehyde levels (MDA)

MDA levels were measured according to the method of Gérard-Monnier et al. with some modifications [65]. Briefly, the reaction mixture contained 6.6 mM N-methyl-2-phenyl-indol (MPI), 0.015 mg/ml butylated hydroxytoluene and 5.55% HCl. The assay was initiated by the addition of sample (1–1.5 mg) and incubated at 45°C for 45 min. To determine the amount of MDA, known concentrations of 1,1,3,3-tetra-ethoxypropane (malondialdehyde bis(diethyl-acetal) (0–20 nmol) were used.

### Electron microscopy

Freshly isolated muscle samples were fixed with 2.5% Glutaraldehyde in PBS and further with 1% Osmium Tetroxide, dehydrated with acetone and embedded in araldite resin. Sections (50 nm) were obtained by using an ultramicrotome (LKB 8800 Ultrotome III). After placing onto a cooper grid, slices were stained for 5 min with uranyl acetate and lead citrate followed by 2 min incubation with lead citrate. Samples were visualized by using a transmission electron microscope (Philips CM10). Ten random images (×5,200) were analyzed to measure the area occupied by mitochondria, the number of mitochondrial structures per unit of area, the area per mitochondria (at least 10 mitochondria per image) and the roundness of each mitochondrial structure by using the ImageJ software version 1.42i (National Institutes of Health, United States).

### Statistics

SigmaStat 3.5 program was used for the statistical analysis and figures were performed by using SigmaPlot 10.0 program (Systat Software Inc). All data are expressed as means ± S.E. For all experiments, 16 mice per group were analyzed. The information obtained from each group was statistically processed pursuant to the most suitable technique for each case. Student t-test or two ways Analysis of Variance (ANOVA) followed by Post-Hoc pairwise multiple comparison procedures (Bonferroni t-test) were performed. The critical significance level α was  = 0.050 and, then, statistical significance was defined as *P*<0.05.
